# Moderate Hyperglycemia-Preventive Effect and Mechanism of Action of *Periplaneta americana* Oligosaccharides in Streptozotocin-Induced Diabetic Mice

**DOI:** 10.3390/nu14214620

**Published:** 2022-11-02

**Authors:** Kaimin Lu, Yufei He, Chuanfang Wu, Jinku Bao

**Affiliations:** 1 Key Laboratory of Bio-Resource and Eco-Environment of Ministry of Education, College of Life Sciences, Sichuan University, Chengdu 610065, China; 2 Pharmacy Research Center, Binzhou Medical University, Yantai 264003, China

**Keywords:** diabetes mellitus (DM), oligosaccharides, inflammation, gut microbiota, oxidative stress, *Periplaneta americana*

## Abstract

*Periplaneta americana* is a kind of medicinal and edible insect, and its oligosaccharides (PAOS) have been reported to exert anti-inflammatory effects by regulating immunity, reducing oxidative stress, and meliorating gut microbiota. We hypothesized PAOS might benefit experimental diabetes mellitus (DM), an inflammatory disease coordinated by both innate and adaptive immunity. This study aimed to evaluate the effect of PAOS on glycemia and its potential mechanisms. Mice model of diabetes was established, and then the potential effects of PAOS was tested in vivo. Here, we found that PAOS triggered a moderate hyperglycemia-preventive effect on DM mice, showing markedly alleviated symptoms of DM, reduced blood glucose, and meliorated functions of liver and pancreas β cell. Deciphering the underlying mechanism of PAOS-improving diabetes, the results revealed that PAOS downregulated the blood glucose level by activating PI3K/AKT/mTOR and Keap/Nrf2/HO-1 pathways, meanwhile inhibiting TLR4/MAPK/NF-κB, Beclin1/LC3, and NLRP3/caspase1 pathways in vivo. Furthermore, analyses of the microbial community intriguingly exhibited that PAOS promoted the communities of bacteria producing short-chain fatty acids (SCFAs), whereas attenuating lipopolysaccharides (LPS)-producing ones that favored inflammatory tolerance. Collectively, balancing the intestinal bacterial communities by PAOS, which favored anabolism but suppressed inflammatory responses, contributed substantially to the glycemia improvement of PAOS in DM mice. Accordingly, PAOS might function as complementary and alternative medicine for DM.

## 1. Introduction

Diabetes mellitus (DM), of any type, is a severe metabolic disease characterized by high blood glucose caused by insulin production defects or inefficient insulin utilization [1]. DM, is related to the reduced quality of life and substantial socioeconomic burden [2]. World Health Organization (WHO) has proposed that 537 million people suffer from diabetes of any type in 2021, and this number will increase to 783 million by 2045 [3].

Type 1 DM is an autoimmune disease resulting from an immune system attack on the islet β cells that produce insulin, leading to an inflammatory process and a lack of insulin in the body [4]. It is usually diagnosed in children and young adults and is determined mainly by host genetics [5]. Furthermore, growing evidence shows that environmental factors, such as diet and intestinal microbiota, play essential roles in the type 1 DM development process [6]. The gut microbiota has been a target to the treatment of type 1 DM [7]. Nowadays, there is considerable interest in searching for high-active compounds from natural products having multiple beneficial effects on glycemia, microbiota, and inflammation, that could help preventing DM or that can be of an additional help for DM treatment.

*Periplaneta americana* is the part of Insecta class, Dictyoptera order, and Blattidae family [8], and is widely distributed in tropical area, which as a medicinal insect is first recorded in an ancient Chinese pharmacopeia “Shen Nong Ben Cao Jing” [9]. The main chemical constituents of *Periplaneta americana* include pheromones, proteins, fatty acids, and esters, amino acids, alkaloids, alkanes, polysaccharides, isofavones, cockroach oil, and peptides, which has been used to treat arthritis, gastric ulcer reverse, fever, carbuncles, pains, and inflammation of the extremities for several hundred years [8,9]. Nature products have great potential for cost-effective benefits with high safety, such as functional oligosaccharides [10]. Our previous study has reported that *Periplaneta americana* oligosaccharides (PAOS) exert anti-inflammatory activity by modulating immune responses, reducing oxidative stress, inhibiting Toll-like receptor 4 (TLR4)/mitogen-activated protein kinase (MAPK)/nuclear factor kappa-B (NF-κB) pathway, preserving intestinal barrier integrity, and regulating gut microbiota with high safety in vivo [11]. However, no investigation has been undertaken to determine the effect of PAOS on glycemia. Thus, it is worth exploring whether PAOS has blood glucose-lowering activity. In this study, a moderate hyperglycemia-preventive effect of PAOS was examined, and its underlying mechanism was investigated in vivo. These findings highlight a new compound from a natural source that could be of additional help in DM therapeutics. 

## 2. Materials and Methods

### 2.1. Materials and Chemicals

The *Periplaneta americana* residues were collected from the Sichuan Gooddoctor-Panxi Pharmaceutical Company (Xichang, Sichuan Province, China).

### 2.2. Preparation of PAOS

The PAOS was obtained following our previous methods [11]. The crude PAOS was extracted by PBS buffer (pH 6.5) in a ratio of 1:10 (*w*/*w*) at 560 w for 60 s in single-mode microwave-assisted synthesis equipment (Monowave 300, Anton Parr, Graz, Austria), and then extracted by 10,000 u papain papaya (Solarbio, Beijing, China) at 65 °C for 4 h. The PAOS was purified by gel filtration chromatography with Sephacry S-100 high resolution column (1.5 × 95 cm, GE, American). The third peak was collected, concentrated, and lyophilized for further research. PAOS consist of 83% glucose, 6% galactose, and 11% xylose with a molecular mass of 1.0 kDa, and the main backbone of PAOS is 1,4-Glcp with 7% branching degree [11].

### 2.3. Animals

Male 6-weeks-old C57BL/6 mice were purchased from Dashuo Laboratory Animal Technology Co. in Chengdu, China. All animal experiments were performed according to the Animal Care and Use Committee of China guidelines. The study was in complete compliance with the National Institutes of Health Guide for the Care and Use of Laboratory Animals and approved by Research Ethics Committee of Sichuan University (NO.20190402001). Prior to the experiments, the mice were housed for at least 1 week at 25 °C under 12 h light/dark cycles with access to standard diet pellets (3.85 kcal/g, about 76% of energy of carbohydrates, 9% of energy of fat, 15% of energy of protein) and water.

### 2.4. Establishment of the DM Model and PAOS Treatment

The mice (n = 40) of DM model were induced by a single intraperitoneal injection of freshly prepared streptozotocin (STZ, 180 mg/kg) dissolved in 0.1 M cold citrate buffer (pH 4.4) [12]. The level of fasting blood glucose (FBG) was tested after 72 h of STZ injection using ACCU-CHEK Performa glucometer (Roche, Basel, Switzerland), and mice were considered diabetic when FBG levels were above 11.1 mmol/L for over one week. After a week, 34 mice successfully developed DM, as confirmed by testing the FBG, 2 mice failed to develop DM, and 4 mice died.

Subsequently, the C57BL/6 mice (n = 8 per group) were randomly divided into control group (Con), model group (M), control + low-dose PAOS group (Con + L), control + high-dose PAOS group (Con + H), control + positive group (Con + P), model + low-dose PAOS group (M + L), model + high-dose PAOS group (M + H), model + positive group (M + P). Glibenclamide is a drug used in type 2 diabetes for increasing the release of insulin from the B cells of the pancreas, so it can be used as a positive drug. The low-dose, high-dose PAOS groups and the positive group were treated orally with 100 mg/kg PAOS, 300 mg/kg PAOS, and 25 mg/kg glibenclamide for 35 days, respectively. Mice of the Con and M groups were given an equal amount of normal saline by intragastric administration for 35 days. The mice were fed with standard diets during the whole experiment. The FBG levels and weight were measured once a week. Mice were sacrificed euthanized with CO_2_ on day 35. The liver was collected and stored at 80 °C for further analysis. Mice feces were collected for intestinal microbiota analysis.

### 2.5. Fasting Serum Insulin Levels

The fasting insulin levels in serum were measured by ELISA kit (Mlbio, Shanghai, China). The homeostasis model of assessment of β-cell function (HOMA-β) was calculated using the following equation [13]:

HOMA − β = 20 × fasting insulin (mU/L)/FBG (mmol/L) − 3.5

### 2.6. Measurement of Cytokines, Serum Parameters and Organ Indexes

Serum was collected after 2000 g centrifugation of blood at 4 °C for 15 min and was used to measure the levels of cytokines and serum parameters. Alanine transaminase (ALT), serum triglyceride (TC), total cholesterol (TG), and low-density lipoprotein cholesterol (LDL-C) levels were determined by the AU5800 Automatic biochemical detector (Beckman, CA, USA). The levels of interleukin (IL)-1β, IL-6, IL-10 and tumor necrosis factor α (TNF-α) in the liver were measured by ELISA kit (Mlbio, Shanghai, China). Spleen and thymus were weighed, and then spleen index and thymus index were calculated using the following equation:

Spleen or thymus index = spleen or thymus weight (mg)/body weight (g)

### 2.7. Measurement of Liver Function and Antioxidant Parameters

The liver tissues were homogenized, and protein concentration was performed by the BCA Protein Assay Kit (NCM, Suzhou, China). The superoxide dismutase (SOD), malondialdehyde (MDA), and glycogen levels in the liver were measured by ELISA kit (Mlbio, Shanghai, China). The levels of aspartate aminotransferase (AST), ALT, albumin (ALB), and total protein (TP) were measured via an AU5800 Automatic biochemical detector (Beckman, CA, USA).

### 2.8. Immunohistochemistry Staining

Pancreas tissues were fixed in 4% paraformaldehyde and embedded in paraffin. The embedded tissues were cut into 4 μm thin sections. Pancreas sections were incubated with antibodies against insulin or glucagon (Abcam, Cambridge, England). Then, colon sections were co-incubated with caspase-3 (Abcam, Cambridge, England). The results of immunohistochemical staining were visualized under a fluorescence microscope equipped with a charge-coupled device camera (Nikon, Tokyo, Japan).

### 2.9. Western Blot

The liver tissues were homogenized, and protein concentration was performed using the BCA Protein Assay Kit (NCM, Suzhou, China). Then, 25 μg proteins were separated with 12% SDS-PAGE gel and then transferred to the PVDF membrane. The membranes were incubated with primary antibodies against Toll-like receptor 4 (TLR4), p65, phosphorylated (P)-p65, p38, P-p38, extracellular signal-regulated kinase (ERK1/2), p-ERK1/2, Jun N-terminal kinase (JNK), P-JNK (Affinity Biosciences, Changzhou, China), phosphatidylinositol 3-hydroxy kinase (PI3K), P-PI3K, protein kinase B (AKT), P-AKT, Foxo1, P-Foxo1, mammalian target of rapamycin (mTOR), P-mTOR, glycogen synthase kinase (GSK3β), P-GSK3β, LC3, Beclin1, Keap1, Nrf2, Heme Oxygenase-1 (HO-1), NOD-like receptor thermal protein domain associated protein 3 (NLRP3), caspase1 or GAPDH (Abcam, Cambridge, England), followed by incubation with secondary antibodies. The signals were developed using an ECL Western blot detection kit (Affinity Biosciences, Changzhou, China) and analyzed by Image J.

### 2.10. Microbial Community Analysis

Mice feces were collected and stored at −80 °C. The DNA of total bacteria in mice feces was extracted with QIAamp^®^ Fast DNA Stool Mini Kit. The V4 region of the 16S rRNA gene was amplified using the barcoded primers. The PCR products were purified using the Qubit 2.0 (Thermo Fisher, Waltham, MA, USA) and sequenced using the Illumina MiSeq platform. All the results were based on sequenced reads and operational taxonomic units (OTUs).

### 2.11. Statistical Analysis

Results were expressed as mean ± SD. The two-way ANOVA test determined differences between groups, followed by Tukey’s post-test using GraphPad Prism version 5. A value of *p* < 0.05 was considered statistically significant and *p* < 0.01 was considered highly significant.

## 3. Results

### 3.1. Effects of PAOS on DM Were Revealed by Body Weight, FBG, Insulin and HOMA-β

**Figure 1:**
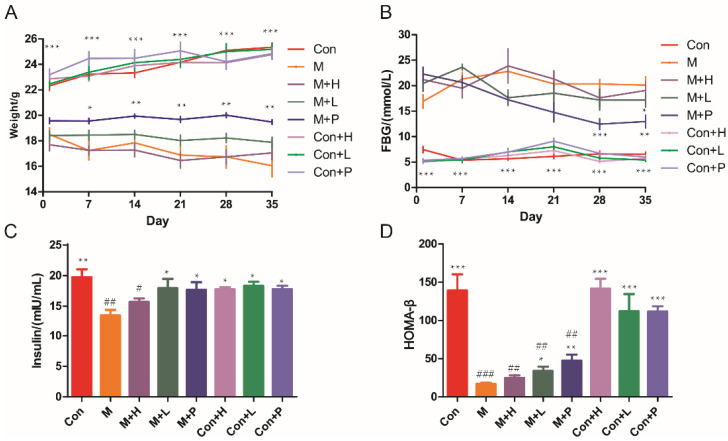
Effects of PAOS on body weight, FBG, insulin, and HOMA-β in TIDM. (**A**) The changes in weight during the experiment; (**B**) the changes in FBG levels during the experiment; (**C**) the level of insulin in serum; (**D**) HOMA-β. Data were expressed as the mean ± SD (n = 8). Con: control group, M: DM model group, M + H: DM + high dose of PAOS group; M + L: DM + low dose of PAOS group, M + P: DM + glibenclamide group; Con + H: control + high dose of PAOS group; Con + L: control + low dose of PAOS group; Con + P: control + glibenclamide group. *** *p* < 0.001, ** *p* < 0.01, * *p* < 0.05 vs. M group; ^###^ *p* < 0.001, ^##^ *p* < 0.01, ^#^ *p* < 0.05 vs. Con group.

The DM mice model was established to investigate the effects of PAOS on DM. STZ treatment induced a marked decrease in body weight (Figure 1A), insulin (*p* = 0.0029, Figure 1C) and HOMA-β (*p* = 0.0002, Figure 1D), and induced an increase in FBG (>11.1 mmol/L, *p* < 0.001, Figure 1B). There was no significant difference in weight between the model group and DM groups treatment with PAOS (Figure 1A). During the administration period, the weight of DM model group tended to be lower, while in the low-dose PAOS or glibenclamide treatment groups the weight did no change and showed lower fluctuation (Figure 1A). On the contrary, the value of FBG showed a lower fluctuation in DM model group, while the value of FBG tended to be (at 14 and 21 days) or was significantly (at 28 and 35 days) lower in glibenclamide group, and the value of FBG rose first and then decreased to be stable in low-dose PAOS group (Figure 1B). Compared to DM model group, low-dose PAOS or glibenclamide treatment were lower by 15.7%, 41.7% for FBG (Figure 1B), were higher by 33.5%, 31.3% for insulin (Figure 1C), and were higher by 98.6%, 177.4% for HOMA-β (Figure 1D), respectively. The control mice treatment with PAOS or glibenclamide groups had no significant difference with control group. These results suggested that PAOS or glibenclamide treatment had a preventive effect on the clinical symptoms of STZ-induced DM.

### 3.2. PAOS Regulated Immune Responses in DM Mice

**Figure 2:**
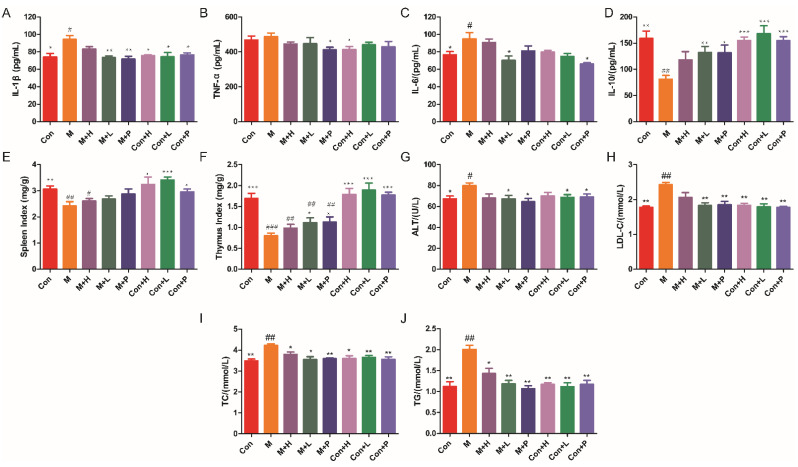
Effects of PAOS on the levels of cytokines in serum, immune organ indexes and serum parameters. Levels of IL-1β (**A**), TNF-α (**B**), IL-6 (**C**), IL-10 (**D**) in serum, spleen index (**E**), thymus index (**F**), and ALT (**G**), LDL-C (**H**), TC (**I**) and TG (**J**) in serum. Data were expressed as the mean ± SD (n = 8). Con: control group, M: DM model group, M + H: DM + high dose of PAOS group; M + L: DM + low dose of PAOS group, M + P: DM + glibenclamide group; Con + H: control + high dose of PAOS group; Con + L: control + low dose of PAOS group; Con + P: control + glibenclamide group. *** *p* < 0.001, ** *p* < 0.01, * *p* < 0.05 vs. M group; ^###^ *p* < 0.001, ^##^ *p* < 0.01, ^#^ *p* < 0.05 vs. Con group.

DM is an autoimmune disease, so the effects of PAOS on the cytokines and immune organs index were explored. The levels of cytokines, including IL-1β, TNF-α, IL-6, and IL-10, were showed in Figure 2A–D. Compared to the control group, the levels of proinflammatory cytokines (IL-1β and IL-6) in serum were markedly higher (*p* = 0.019, *p* = 0.0362), and the level of anti-inflammatory cytokine (IL-10) in serum was lower in DM model group (*p* = 0.0013). As shown in Figure 2A–D, in low dose PAOS or glibenclamide treatment group, the levels of IL-1β (*p* = 0.0021, *p* = 0.003) were lower, and the level of IL-10 (*p* = 0.0056, *p* = 0.014) were higher in DM group. As regards to the level of IL-6, glibenclamide treatment did not conduct to a different value (*p* = 0.2035), while low dose PAOS treatment led to a significant lower value (*p* = 0.0236). As depicted in Figure 2E, F, the spleen and thymus indexes were lower by 20.9% and 52.7% in DM, respectively. Low dose PAOS or glibenclamide administration were higher by 39.4% or 41.3% of thymus index. There was no significant difference between the control group of treatment with PAOS or glibenclamide and the control group. These findings indicated that PAOS relieved symptoms of DM by preventing inflammation to occur and immunomodulatory effects.

### 3.3. PAOS Reduced Serum Parameterslevels

The serum parameters levels were displayed in Figure 2G–J. In DM, the serum parameters, including ALT, LDL-C, TC, and TG, were markedly higher (*p* = 0.0282, *p* = 0.0011, *p* = 0.0027, *p* = 0.004) than control group mice. After administration of low dose PAOS or glibenclamide, the levels of ALT (Figure 2G), LDL-C (Figure 2H), TC (Figure 2I), and TG (Figure 2J) were lower by 15.7% or 19.1%, 24.5% or 23.5%, 15.8% or 14.9 and 40.9% or 46.8%, respectively, compared to DM model mice. PAOS or glibenclamide had no significant effects on serum parameters levels in control mice. These results suggested that PAOS eased DM symptoms by reducing serum parameters levels.

### 3.4. PAOS Benefited Liver Function and Antioxidant Parameters in DM Mice

**Figure 3:**
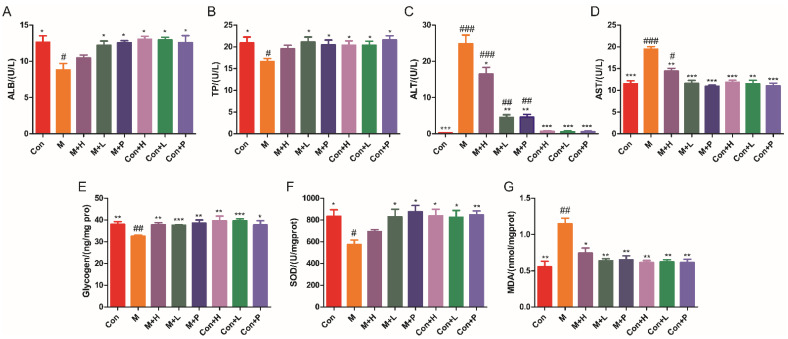
PAOS benefited the liver function and oxidative stress parameters. Levels of ALB (**A**), TP (**B**), ALT (**C**), AST (**D**), glycogen (**E**), SOD (**F**), and MDA (**G**) in liver. Data were expressed as the mean ± SD (n = 8). Con: control group, M: DM model group, M + H: DM + high dose of PAOS group; M + L: DM + low dose of PAOS group, M + P: DM + glibenclamide group; Con + H: control + high dose of PAOS group; Con + L: control + low dose of PAOS group; Con + P: control + glibenclamide group. *** *p* < 0.001, ** *p* < 0.01, * *p* < 0.05 vs. M group; ^###^ *p* < 0.001, ^##^ *p* < 0.01, ^#^ *p* < 0.05 vs. Con group.

The liver is an important site where glucose is converted into glycogen. The effects of PAOS on the parameters of liver function and antioxidant were explored. As shown in Figure 3A–D, compared with control mice, the liver function parameters (ALB, TP, ALT and AST) were abnormal in DM, which suggested that liver function in mice with diabetes was abnormal. The levels of ALB (Figure 3A) and TP (Figure 3B) were lower by 30.2% and 20.7%, and the contents of ALT (Figure 3C) and AST (Figure 3D) were higher by 98.5-fold and 69.2% in DM, respectively. The mice in treatment with low-dose PAOS or glibenclamide showed higher ALB (*p* = 0.0329, *p* = 0.0169) and TP (*p* = 0.028, *p* = 0.0404), and dramatically lower ALT (82% or 80.5%, respectively) and AST (40.4% or 44%, respectively) than model group. As shown in Figure 3E, liver glycogen level was lower in DM (*p* = 0.0028), while it was higher in the PAOS or glibenclamide-treated DM groups (*p* = 0.0019, *p* = 0.0004, *p* = 0.0027). Compared with the DM group, the level of antioxidant enzyme SOD in PAOS or glibenclamide treatment group was significantly higher (Figure 3F, *p* = 0.0589, *p* = 0.0353, *p* = 0.0128). On the contrary, the level of MDA (maker of oxidative stress) in liver tissues in low-dose PAOS or glibenclamide treatment group was lower by 44.3% or 43.5%, respectively (Figure 3G). However, PAOS or glibenclamide had no significant effect on liver function and antioxidant parameters of control mice. These findings demonstrated that PAOS or glibenclamide had potential efficacy of improving liver function and reducing oxidative stress.

### 3.5. PAOS Improved Islet β Cell Function and Downregulated Its Apoptosis in DM Mice

**Figure 4:**
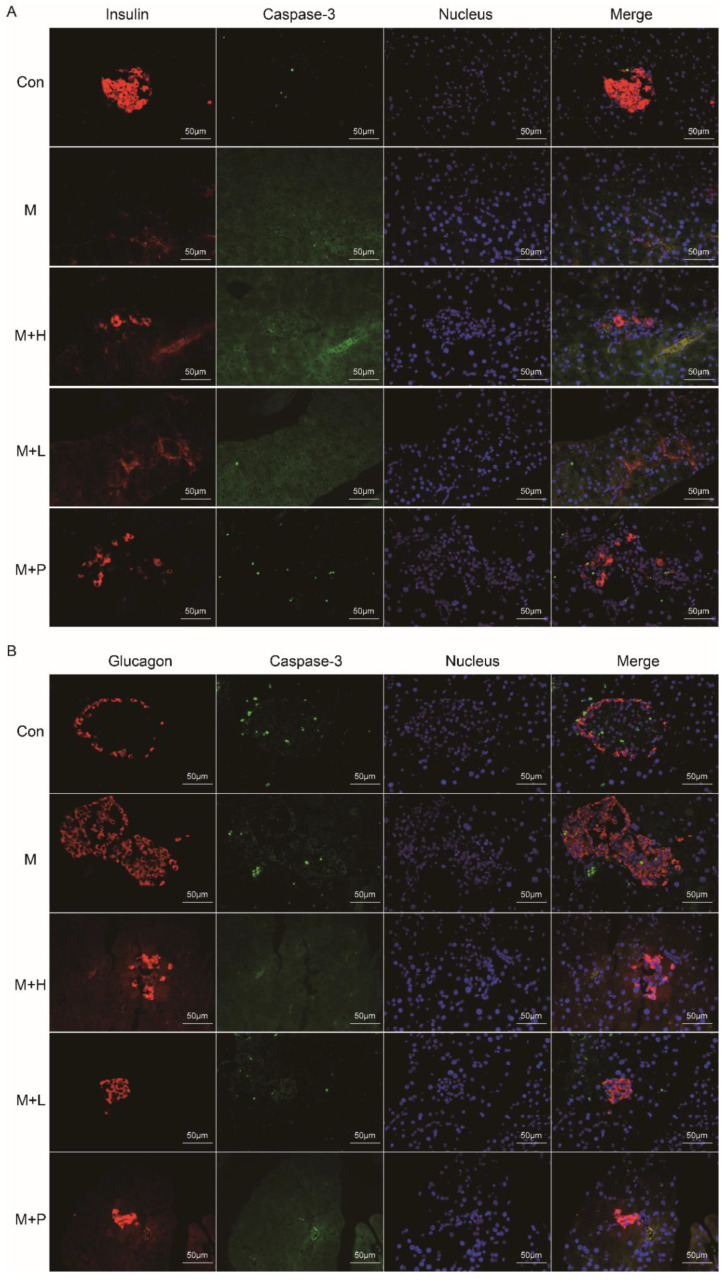
PAOS improved islet β cell function and downregulated its apoptosis in the pancreas. (**A**) The effects of PAOS on insulin (β-cells) and apoptosis of β cells in the pancreas. The red fluorescence was insulin, the green fluorescence was caspase-3, the blue fluorescence was the nucleus, and the Merge fluorescence was the overlap of the three kinds of fluorescence. (**B**) The effects of PAOS on glucagon (α cells) and apoptosis of α cells in the pancreas. The red fluorescence was glucagon, the green fluorescence was caspase-3, the blue fluorescence was the nucleus, and the Merge fluorescence was the overlap of the three kinds of fluorescence. Con: control group, M: DM model group, M + H: DM + high dose of PAOS group; M + L: DM + low dose of PAOS group, M + P: DM + glibenclamide group.

The pancreas function was detected by immunohistochemistry. As shown in Figure 4A,B, the insulin-positive β-cells and glucagon-positive α-cells were analyzed, respectively. Compared to the control group, the immunofluorescence staining exhibited that the insulin expression (β cells) was decreased and glucagon expression (α cells) were increased in pancreatic islets of diabetic mice. PAOS or glibenclamide treatment prevented these changes, the insulin expression β-cells were elevated and glucagon expression α-cells were reduced in diabetic mice. Moreover, the caspase-3 positive cells in islet β- and α-cells were analyzed (Figure 4A,B). In diabetes mice, the apoptosis-positive of islet β-cells was increased. After administration of PAOS or glibenclamide, the apoptosis-positive of islet β-cells was diminished in diabetic mice. These results suggested that PAOS or glibenclamide could rise insulin expression via inhibiting the apoptosis of islet β-cells.

### 3.6. PAOS Regulated Signal Pathways in the Livers of TIDM Mice

**Figure 5:**
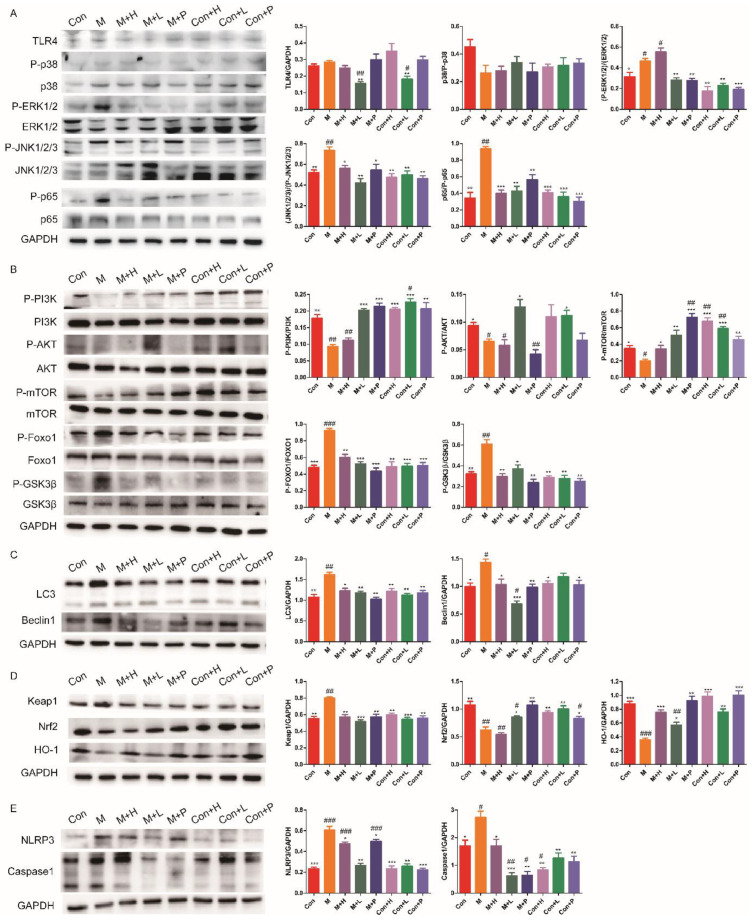
Effects of PAOS on the related signal pathways in DM mice. The effects of PAOS on the TLR4/MAPK/NF-κB (**A**), PI3K/AKT (**B**), Beclin1/LC3 (**C**), Keap1/Nrf2/HO-1 (**D**) and NLRP3/caspase1 (**E**) pathways in the liver. Con: control group, M: DM model group, M + H: DM + high dose of PAOS group; M + L: DM + low dose of PAOS group, M + P: DM + glibenclamide group; Con + H: control + high dose of PAOS group; Con + L: control + low dose of PAOS group; Con + P: control + glibenclamide group. *** *p* < 0.001, ** *p* < 0.01, * *p* < 0.05 vs. M group; ^###^ *p* < 0.001, ^##^ *p* < 0.01, ^#^ *p* < 0.05 vs. Con group.

TLR4/MAPK/NF-κB pathway has been shown to contribute to the inflammatory response. The expressions of key components in the TLR4/MAPK/NF-κB signaling pathway are shown in Figure 5A. Compared with the control group, the levels of some important proteins (including, P-p38, P-ERK1/2, P-JNK1/2/3 and P-p65) in TLR4/MAPK/NF-κB pathway were higher (*p* = 0.0722, *p* = 0.0329, *p* = 0.0077, *p* = 0.0012) in DM. Low dose of PAOS inhibited the expression levels of TLR4 (*p* = 0.001), and PAOS or glibenclamide significantly inhibited the phosphorylation of ERK1/2, JNK, and p65. There was no significant effect on TLR4/MAPK/NF-κB signaling pathway in control mice treated with PAOS or glibenclamide. These results revealed the anti-inflammatory effects of PAOS via suppressing TLR4/MAPK (ERK1/2 and JNK)/NF-κB pathway. The glucose metabolism PI3K/AKT/mTOR signaling pathway was remarkably downregulated in DM (Figure 5B). Moreover, the protein expressions of P-Foxo1 and P-GSK3β were upregulated in the livers of DM mice (Figure 5B). The treatment of PAOS or glibenclamide normalized the glucose metabolism, gluconeogenesis, and glycogen synthesis (Figure 5B), suggesting that PAOS or glibenclamide reduced the blood glucose via activating PI3K/AKT/mTOR pathway. PAOS or glibenclamide had no significant effects on glucose metabolism in control mice. Expressions of key proteins in the autophagy signaling pathway are depicted in Figure 5C. The levels of LC3 and Beclin1 were significantly higher in DM (*p* = 0.0036, *p* = 0.0105), PAOS or glibenclamide could downregulate the expression of LC3 and Beclin1. PAOS or glibenclamide had no significant effects on the level of LC3 and Beclin1 in control mice. As shown in Figure 5D, compared with the control group, expression of Keap1, Nrf2 and HO-1 (the key proteins in the antioxidative pathway) were abnormal in DM. After administration of PAOS or glibenclamide, the level of Keap1 was significantly downregulated (*p* = 0.0024, *p* = 0.0005, *p* = 0.0029), and the expressions of Nrf2 and HO-1 were upregulated (*p* = 0.0188, *p* = 0.0057, *p* = 0.007; *p* = 0.0007, *p* = 0.0104, *p* = 0.0012) in DM. These findings confirmed the previous results of reducing oxidative stress. The levels of important proteins in the pyroptosis signaling pathway, including NLRP3 and caspase1, were analyzed in Figure 5E. The expression of NLRP3 and caspase1 were remarkably higher (*p* = 0.0005, *p* = 0.0267), PAOS or glibenclamide downregulated the levels of NLRP3 and caspase1 (*p* = 0.0243, *p* = 0.001, *p* = 0.0475; *p* = 0.0336, *p* =0.001, *p* = 0.0013) in DM, suggesting that PAOS or glibenclamide downregulated the pyroptosis signaling pathway.

### 3.7. PAOS Regulated the Gut Microbiota in Control Mice

**Figure 6:**
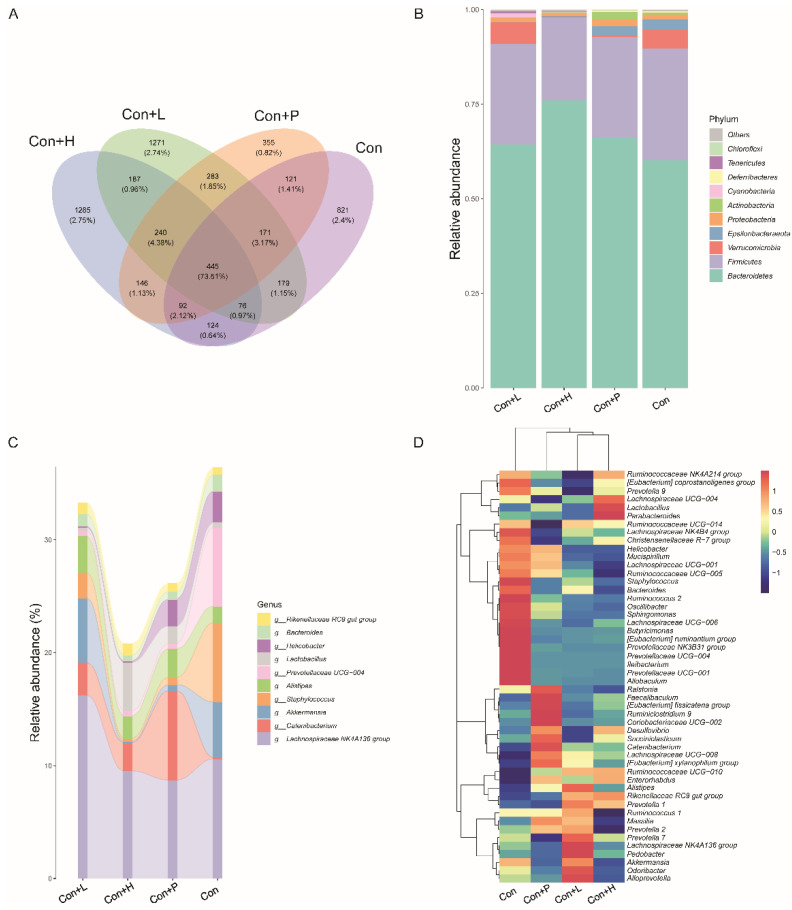
Effects of PAOS on gut microbiota at phylum and genus levels in control mice. (**A**) Venn chart; (**B**) the relative abundance at the phylum level. (**C**) The analysis of sankeyplot of high abundance genus; (**D**) the heap maps of relative abundance at the genus level. Con: control group; Con + H: control + high dose of PAOS group; Con + L: control + low dose of PAOS group; Con + P: control + glibenclamide group.

**Figure 7:**
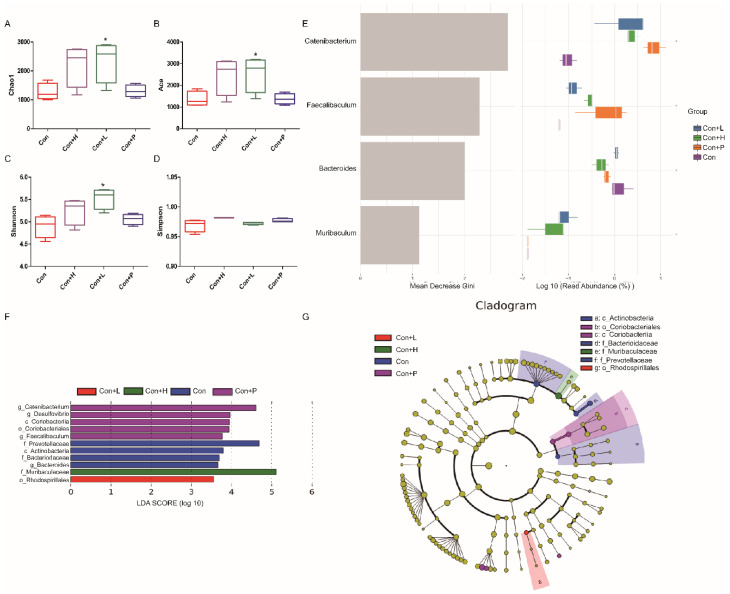
Effects of PAOS on the alpha diversity and the biomarkers of gut microbiota in control mice. Chao1 (**A**), Shannon (**B**), Simpson (**C**), Faith’s PD (**D**) of gut microbiota, respectively; the random forest analysis (**E**); LDA score of Lefse (**F**); Cladogram of Lefse (**G**). Con: control group; Con + H: control + high dose of PAOS group; Con + L: control + low dose of PAOS group; Con + P: control + glibenclamide group. * *p* < 0.05 vs. Con group.

To investigate PAOS’s role on gut microbiota in control mice, the relative abundance at phylum level and genus level, sankeyplot of high abundance genus are shown in Figure 6A–E. Venn diagram (Figure 6A) shows the logical connection between each group, and shows that 73.51% of the intestinal microbiota was overlapping. As shown in Figure 6B, compared with control group, the level of Epsilonbacteraeota in all PAOS treatment control group was lower, and the levels of Verrucomicrobia in the low-dose PAOS and glibenclamide group were lower. Compared with the control group, the level of Catenibacterium was higher, and the levels of Staphylococcus, Prevotellaceae UGG-004, Helicobacter, and Bacteroides were lower in all PAOS or glibenclamide treatment control group (Figure 6C, D). The alpha diversity represented by Chao1, ACE, Simpson and Shannon is displayed in Figure 7A–D. Compared with the control group, the indexes of Chao1, Ace and Shannon were significantly higher (*p* = 0.0331, *p* = 0.0383, *p* = 0.0102) in the low-dose PAOS treatment control group, suggesting low-dose PAOS increased the diversity of gut microbiota in standard mice. To explore the biomarkers of gut microbiota among each group, random forest and LEFse analysis were depicted in Figure 7E–G. The microbiota such as *Catenibacterium*, *Faecalibacterium*, *Bacteroides*, and *Muribaculum* with high values of mean decrease Gini, was influential in the classifying groups (Figure 7E). As seen in Figure 7F,G, the prominent and important intestinal microbiota in the gut were *Actinobacteria*, *Prevotellaceae*, and *Bacteroidaceae* in the control group; the prominent and crucial intestinal microbiota in the gut was *Muribaculum* in the high-dose PAOS treatment standard mice group; the prominent and essential intestinal microbiota in the gut was *Rhodospirillales* in low-dose PAOS treatment control mice group; the prominent and vital intestinal microbiota in the gut was *Coriobacterlia* in glibenclamide treatment control mice group.

### 3.8. PAOS Ameliorated on Gut Microbiota in DM Mice

**Figure 8:**
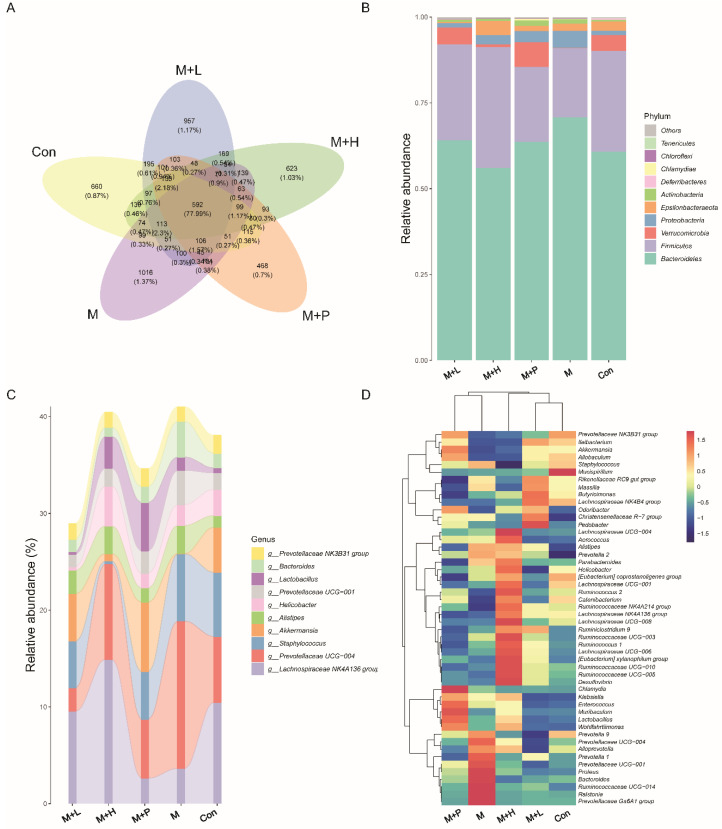
Effects of PAOS on gut microbiota at phylum and genus level in DM. (**A**) Venn chart; (**B**) The relative abundance at the phylum level. (**C**) The analysis of sankeyplot of high abundance genus; (**D**) The heap maps of relative abundance at the genus level. Con: control group, M: DM model group, M + H: DM + high dose of PAOS group; M + L: DM + low dose of PAOS group, M + P: DM + glibenclamide group.

**Figure 9:**
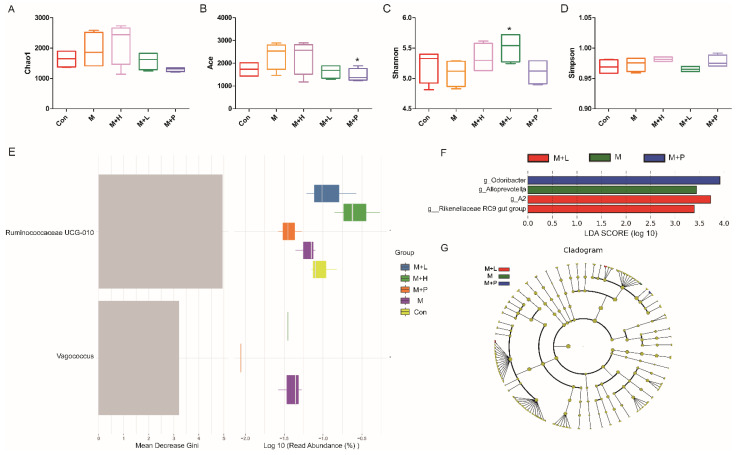
Effects of PAOS on the alpha diversity and the biomarkers of gut microbiota in DM. Chao1 (**A**), Shannon (**B**), Simpson (**C**), Faith’s PD (**D**) of gut microbiota, respectively; the random forest analysis (**E**); LDA score of Lefse (**F**); Cladogram of Lefse (**G**). Con: control group, M: DM model group, M + H: DM + high dose of PAOS group; M + L: DM + low dose of PAOS group, M + P: DM + glibenclamide group. * *p* < 0.05 vs. M group.

**Figure 10:**
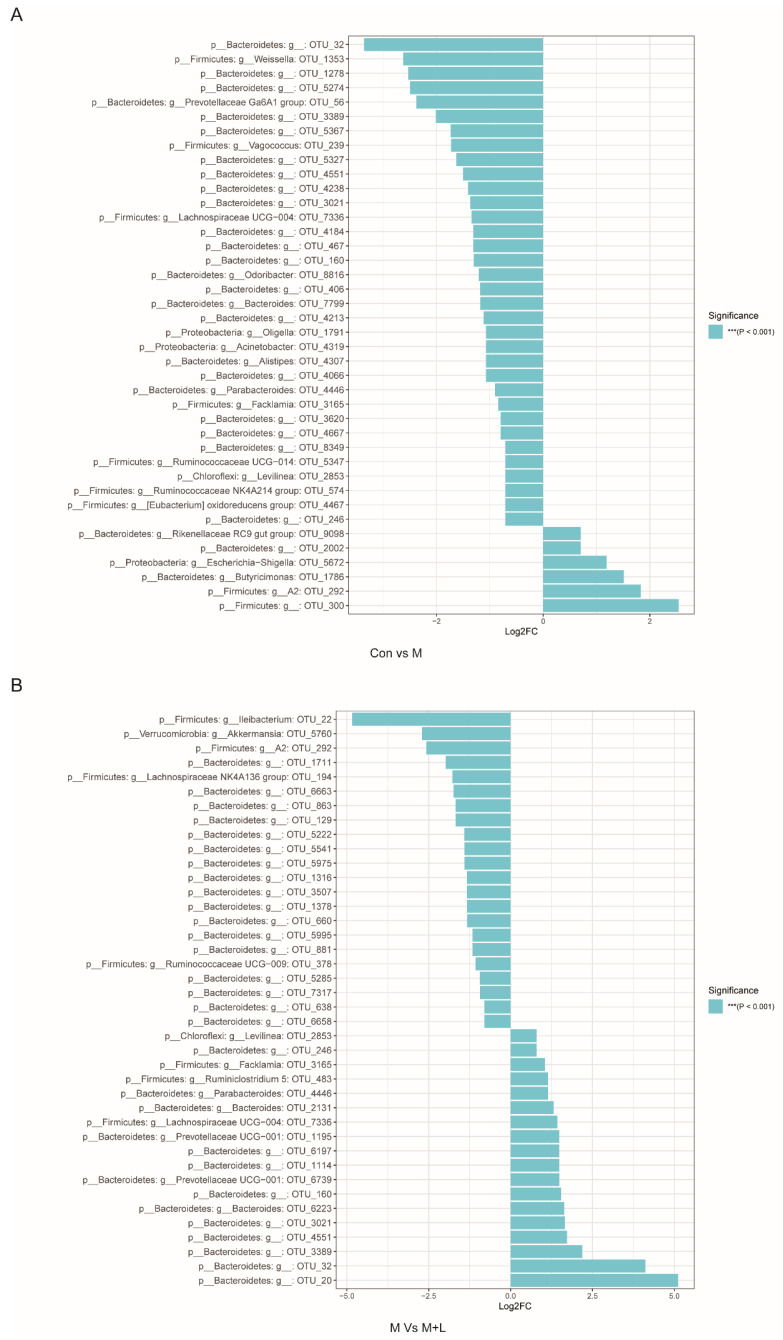
MetagenomeSeq analysis of gut microbiota in DM. (**A**) MetagenomeSeq analysis of Con VS M group; (**B**) MetagenomeSeq analysis of M vs. M + L group. Con: control group, M: DM model group; M + L: DM + low dose of PAOS group.

To explore the effects of PAOS on gut microbiota in DM mice, the relative abundance at phylum level and genus level, sankeyplot of high abundance genus are shown in Figure 8A–D. Venn diagram (Figure 8A) showed 77.99% of the intestinal microbiota in each group was overlapping. As shown in Figure 8B, compared with the control group, the levels of Bacteroidetes, Proteobacteria, and Actinobacteria were higher, the levels of Firmicutes and Verrucomicrobia were lower, and the ratio of Firmicutes/Bacteroidetes was lower in DM mice. The PAOS could elevate the level of Firmicutes, Verrucomicrobia, and the ratio of Firmicutes/ Bacteroidetes, and reduce the levels of Bacteroidetes and Proteobacteria in DM mice. As seen in Figure 8C,D, compared with the control group, the levels of *Prevotellaceae UGG-004*, *Prevotellaceae UGG-001*, *Prevotella1*, *Prevotellaceae Ga6A1 group*, *Ralstonia Proteus*, and *Bacteroides* were higher, and the levels of *Akkermansia*, *Allobaculum*, *Ruminococcus2*, *Ileibacterium*, and *Odoribacter* were lower in DM mice. The treatment of PAOS or glibenclamide prevented such changes. The alpha diversity represented by Chao1, ACE, Simpson, and Shannon were displayed in Figure 9A–D. Compared with the control group, the indexes of Chao1, Ace, and Shannon had no significant difference in DM group. The low-dose PAOS treatment elevated the index of Shannon (*p* = 0.0438) in DM. To explore the biomarkers of gut microbiota among each group, the random forest and LEFse analyses were depicted in Figure 9E–G. The microbiota, such as *Ruminococcaceae UGG-010* and *Vagococcus* with high values of mean decrease Gini, were crucial in classifying groups (Figure 9E). As seen in Figure 9F,G, the prominent and influential intestinal microbiota in the gut was *Alloprevotella* in the DM group; the prominent and essential intestinal microbiota in the gut were *A2* and *Rikenellaceae RC9 gut group* in the low-dose PAOS treatment DM group; the prominent and vital intestinal microbiota in the gut was *Odoribacter* in glibenclamide treatment DM group. The Figure 10A,B showed that compared with control group, the amount of Bacteroidetes was increased in DM, and the low-dose of PAOS reduced the amount of Bacteroidetes. These results indicated that PAOS improved the gut microbiota in DM mice.

## 4. Discussion

STZ is the most widely used chemical drug for establishing the diabetes mice or rat model, which causes pancreatic islet damage and thus reduces insulin secretion [14]. Some natural products exert antidiabetic effects via several mechanisms, including promoting insulin secretion, inhibition of carbohydrate metabolizing enzymes and regeneration of β-cells, such as alkaloids, flavonoids, phenolic acids, saponins, and terpenoids [15]. Some studies have reported that functional oligosaccharides have antidiabetic activity, such as fructo-oligosaccharides (FOSs) [16], mannan-oligosaccharides (MOSs) [17], and chito-oligosaccharides (COSs) [18]. In this study, we demonstrated that PAOS improved glucose-lipid metabolic disorder and glycemic control to mitigate risks of DM through modulation in inflammation, oxidative stress, and gut microbiota.

DM is considered a β-cell-mediated pro-inflammatory state induced by innate and adaptive immunity. TLR4/MAPK/NF-κB pathway is the primary mediator of inflammatory responses, which plays a crucial role in the immune and inflammatory response [19], which regulates the release of many proinflammatory cytokines and then causes β-cell death in DM [20]. Pyroptosis is a form of pro-inflammatory cell death [21], induced by the classic pathway (depended on caspase 1) or non-classic pathway (depended on caspase 4/5/11). PAOS inhibited the expression of TLR4, then down-regulated the phosphorylation of ERK and JNK in the MAPK family and p65 in the NF-κB pathway, which reduced the expression of proinflammatory cytokines. Moreover, PAOS suppressed the classic pyrolysis pathway. These findings indicated that PAOS relieved DM by reducing inflammation.

Significantly lack of insulin and high blood glucose are the features of DM. HOMA-β is the usual assessment of islet β-cell function that releases insulin [22]. Insulin regulates glucose homeostasis via promoting glucose transport into muscle and adipose cells, while binding to the insulin receptor and then actives insulin cascade [23]. FOSs increased insulin secretion and improved β-cell function in DM mice [24]. PAOS also recovered the HOMA-β via reducing the apoptosis of islet β-cell and then elevating insulin secretion. The activation of the insulin pathway, PI3K/AKT/mTOR pathway, reduces blood glucose by inhibiting gluconeogenesis and stimulating glycogen synthesis [23,25]. PAOS down-regulated the PI3K/AKT pathway, then downregulated the phosphorylation of GSK-3β and FOXO1, which suggested that PAOS suppressed gluconeogenesis and promoted glycogen synthesis. The liver glycogen is crucial for maintaining blood glucose levels [14]. PAOS increased liver glycogen levels by improving liver function parameters (ALB, TP, ALT and AST). Aberrant blood lipid metabolism is critical for DM progression [26]. Oligo-N-acetylglucosamine can significantly reduce the lipid profile, including TG, TC, and LDL [27]. PAOS reduced the levels of ALT, LDL-C, TC, and TG. These results showed that PAOS ameliorated symptoms of DM via activating insulin cascade, reducing the blood lipid and improving liver function.

Hyperglycemia induces reactive oxygen species production and leads to oxidative stress in DM. Moreover, oxidative stress can lead to cell autophagy death and inflammation, so lowering the oxidative injury is essential for DM treatment [28,29]. COSs exhibits oxidation resistance bioactivities in alloxan-induced DM [30]. Keap1/Nrf2 pathway is an endogenous antioxidant signaling pathway. PAOS unbound Nrf2 with Keap1 in the cytoplasm, then activated Nrf2 was transported to the nucleus and stimulated the expression of antioxidase HO-1. Meanwhile, PAOS increased the level of SOD and decreased the MDA level. Beclin1, LC3, and mTOR play essential roles in autophagy. PAOS elevated the phosphorylation of mTOR and then down-regulated the expression of Beclin1 and LC3, which suggested that PAOS inhibited autophagy pathway. These findings suggested that PAOS reduced blood glucose by lowering oxidative stress and suppressing autophagy.

Interactions of gut microbiota components play key roles in immune and inflammatory functions [7]. Studies have shown that the composition of major microbial groups differs between healthy individuals and those with DM. The ratio of Firmicutes to Bacteroidetes (F/B) may be the early diagnostic marker of DM [31]. PAOS elevated the ratio of F/B in DM mice. The LPS derived from microbiota in the gut can promote the release of proinflammatory cytokines and damage pancreatic β-cell function [32,33]. The study has reported that hosts with DM have higher LPS and LPS-producing bacteria than healthy hosts. LPS may act as the link between inflammation, gut microbiota, and DM [34]. Functional oligosaccharides could reduce LPS and then reduce inflammation, such as FOS [24]. PAOS reduced the amounts of LPS producing bacteria, such as Bacteroidetes and Proteobacteria, and then inhibited the downstream TLR4/MAPK/NF-κB pathway in DM mice. Bacterial-producing short-chain fatty acids (SCFAs, such as butyrate and acetate) were reduced in DM [35]. SCFAs play an essential role in metabolic, immune response, and inflammatory diseases, such as DM [36]. Functional oligosaccharides improve glucose metabolism by regulating intestinal microbiota and increasing the secretion of SCFAs [37]. PAOS increased the amounts of SCFAs producing bacteria, such as *Odoribacter*, *Ruminococcus2*, and *Allobaculum*, which regulated blood glucose by decreasing inflammation. Moreover, PAOS increased the *Akkermansia* that could reduce the risk of diseases, such as obesity, diabetes, and inflammation. All these results indicated that PAOS regulated blood glucose by improving the structure of gut microbiota and downregulated the TLR4/MAPK/NF-κB pathway.

In conclusion, PAOS exerted a hyperglycemia-reducing effect by reducing inflammation and oxidative stress, improving pancreas function, enhancing immunity, and regulating gut microbiota. Inflammation, immune, oxidative stress, and gut microbiota could form an interactive network to regulate blood glucose in DM mice. All these results may pave a new way for developing effective drugs with high safety that can be applied in DM treatment in the future.
